# Effects of dietary chlorogenic acid on ileal intestinal morphology, barrier function, immune factors and gut microbiota of broilers under high stocking density stress

**DOI:** 10.3389/fphys.2023.1169375

**Published:** 2023-04-14

**Authors:** Yu-Qian Li, Yi Zhang, Dong-Ying Bai, Yan-Hao Liu, Xiang-Long He, Koichi Ito, Ke-Xin Liu, Hai-Qiu Tan, Wen-Rui Zhen, Cai Zhang, Bing-Kun Zhang, Yan-Bo Ma

**Affiliations:** ^1^ Department of Animal Physiology, College of Animal Science and Technology, Henan University of Science and Technology, Luoyang, China; ^2^ Innovative Research Team of Livestock Intelligent Breeding and Equipment, Longmen Laboratory, Luoyang, China; ^3^ Henan International Joint Laboratory of Animal Welfare and Health Breeding, College of Animal Science and Technology, Henan University of Science and Technology, Luoyang, China; ^4^ Department of Food and Physiological Models, Graduate School of Agricultural and Life Sciences, The University of Tokyo, Ibaraki, Japan; ^5^ State Key Laboratory of Animal Nutrition, Department of Animal Nutrition and Feed Science, College of Animal Science and Technology, China Agricultural University, Beijing, China

**Keywords:** high stocking density stress, chlorogenic acid, antioxidant capacity, ileac barrier function, microbial community, broilers

## Abstract

**Aims:** The purpose of this research was to assess the effect of chlorogenic acid (CGA) in the diet on ileac structure, barrier function, immunological state, and microbial profile of broiler chickens in a high stocking density (HD) environment.

**Methods:** Four hundred and seventy-six male AA broiler chickens were randomly split into four groups, two with a normal stocking density (ND) of fourteen birds per m^2^ and two with a high stocking density of twenty-two birds per m^2^. Each of the treatments consisted of five replicates. One of the two ND and HD groups received the usual feed, while the other two were given at 1.5 g/kg CGA as part of their dietary regimen.

**Results:** The ND CGA group showed a greater increase in villus height and villus height/crypt depth compared to the ND group at 35 and 42 days. The HD group experienced a greater elevation in villus height due to CGA supplementation than the HD group across days 28, 35, and 42. At day 42, the HD group saw a decline in *OCLN* and *ZO-1* mRNA expression in the ileum, but CGA was able to restore them. The HD group experienced a greater rise in *OCLN* mRNA than the control HD group when supplemented with CGA. The expression of *TNF-α, IL-1β*, and *IL-6* in the ileum was higher in the HD group, and CGA supplementation enhanced this effect. The HD group experienced a greater rise in *IL-10* mRNA expression than the control group following the administration of CGA. The HD group showed reduced alpha diversity and an increase in detrimental microbes such as *Turicibacter* and *Shigella* in the gut compared to the ND group, while the HD CGA group saw a reduction in *Turicibacter, Shigella*, and other harmful microbes. These findings reveal that HD stress suppressed the growth of ileac villi, decreased the expression of tight-junction genes, amplified the expression of inflammatory genes, and disturbed the gut microbiota, ultimately leading to increased intestinal permeability.

**Conclusion:** We conclude that when chickens are given dietary CGA, the disruption of the ileac barrier and increased oxidative damage and inflammation due to HD stress are reduced, which increases ileac integrity and the presence of beneficial intestinal bacteria.

## 1 Introduction

In recent years, the poultry industry has seen a major increase in production of broilers worldwide to meet the demands of a growing population. This extensive development has not only improved quality of life, but has had a substantial positive effect on national economies (Li et al., 2019). As broilers have become more important as a meat source, it would be more cost-effective to increase the stocking density (SD) in order to produce more meat per unit area (Thaxton et al., 2006). Nevertheless, there is a growing emphasis on animal health and welfare concerns, which are closely linked to SD (Vanhonacker et al., 2009; Wang et al.,2019). SD varies from country to country with 45–54 kg/m^2^ being the norm in the Netherlands, 40 kg/m^2^ in the United Kingdom, 41.5 kg/m^2^ in the United States, and 30–36 kg/m^2^ in Switzerland (Nasr et al., 2021). A recommended SD, in consideration of animal welfare, is thought to be beneficial for broiler production and high product quality (Simitzis et al., 2012). High stocking density (HD) has been shown to cause oxidative stress and has been linked to diminished production in broilers, as well as an increased likelihood of health problems (Dozier et al., 2005). Consequently, many strategies have been implemented to improve production of broiler chickens under HD (Abd El-Hack et al., 2020; Sugiharto, 2022). It has been widely accepted that dietary supplementation with certain antioxidants is a viable and convenient solution to reduce HD stress.

As the site in the digestion of starches and fats, the ileum harbors a highly varied microbial population, making intestinal homeostasis a critical element in maintaining the health of broilers (Stanley et al., 2014). HD stocking can cause oxidative stress (OS), which can damage ileal structures by shortening the villi, deepening the recesses, and depleting mucosal epithelial cells, leading to severe disruption of ileal integrity (Tan et al., 2010). Research has demonstrated that OS can dramatically decrease the expression of *occludin* (OCLN), *claudin-1* (CLDN1), and *zonula occludens-1* (ZO-1) in the broiler intestine (He et al., 2016; Zhang et al., 2017), along with an increase in the expression of *interleukin-6* (IL-6) (Quinteiro-Filho et al., 2017) and *tumor necrosis factor-α* (TNF-α) (He et al., 2019) in the intestinal mucosa, and a decrease in *interleukin-10* (IL-10) mRNA expression in the intestine (Quinteiro-Filho et al., 2017). Despite this, it is still unclear if OS caused by HD has a negative effect on ileal homeostasis.

Chlorogenic acid (CGA), a bioactive dietary polyphenol, is esterified by quinic acid and caffeic acids, which are found in green coffee bean extract (Tajik et al., 2017). CGA is widely used in many Chinese herbal medicines, derived from botanical sources like *Dendranthema grandiflora* (*L. chrysanthemum*), *F. lonicerae* (*Flos lonicerae*) and *Eucommia ulmoides* (*E. ulmoides*) (Leiss et al., 2009). It has been documented that CGA has a range of pharmacological effects in animals, which can augment development, strengthen immunity, and modify the gut microbiota by enhancing bioactivities such as antioxidant (Liang and Kitts, 2015), anti-inflammatory (Vukelić et al., 2018), antibacterial (Zhang et al., 2020), and antiviral (Gamaleldin Elsadig Karar et al., 2016), and by regulating lipid metabolism (Sung et al., 2015). In light of current regulations to limit the generation of antimicrobial resistance by eliminating addition of antibiotics to feed, as well as restrictions on resistance in livestock and poultry breeding, the future of animal husbandry appears to be returning to the use of safe natural plant products with similar medicinal properties (Jiang and Xiong, 2016; Vizzier Thaxton et al., 2016).

Our prior research has demonstrated that chickens experienced a decrease in weight gain and feed intake due to HD stress at 28, 35, and 42 days. Despite this, administering 1.5 g/kg of CGA proved to be effective in enhancing both body weight and average daily gain in the HD chickens. These data presented points to the potential of CGA supplementation in chicken diets to counteract the effects of HD stress on chicken productivity (Liu et al., 2023). Consequently, we forecast that CGA boosts efficacy from HD stress by fortifying ileum wellness and augmenting advantageous bacteria. The main goal of this study was to assess the influence of supplementing the feed with CGA on the ileal structure, protective capability, immune system, and gut bacteria of broilers under HD stress conditions at 28, 35, and 42 days of age.

## 2 Materials and methods

### 2.1 Statement of ethical treatment

The Care and Use of Experimental Animals Committee of the Henan University of Science and Technology (HUST) (AW20602202-1-3) gave its consent to the experimental protocol of this study in 2020. The experiments conformed to their regulations for the humane treatment of animals.

### 2.2 Animals and experimental design

Four hundred and seventy-six healthy one-day-old male Arbor Acres broiler breeders were obtained from the Henan Quanda Poultry Breeding Co., Hebei, China, and experiments were carried out at the Animal Research Unit of HUST. The chickens were inspected upon arrival to detect illness or physical problems. Chickens were housed with an automated system for ventilation, temperature, humidity, and illumination regulation. At the beginning of the experiments, the temperature of the feeding room was held at 33°C ± 1°C for 1 week, then gradually lowered by 1°C-2°C per week until it reached a final temperature of 25°C ± 2°C by the 42nd day. The room humidity was maintained at 60%–70 %, and lights were kept on for 23 h daily and turned off from 7 to 8 p.m. On the seventh day, five replicates each of healthy broilers, with an average weight of 138.5 ± 2.2 g, were randomly distributed among four different experimental treatments: normal stocking density of 14 birds/m^2^ (ND), high stocking density of 22 birds/m^2^ (HD), and dietary supplementation with CGA at 1.5 g/kg in the ND CGA and HD CGA groups. To guarantee that the density stayed the same for each sampling date, three extra sets were prepared for every density group.

### 2.3 Diet composition and CGA supplementation

The main ration for broilers consisted of the corn-soybean meal pellet feed, with the nutritional information listed in Table 1. The supplemental CGA (98% pure) was provided by Changsha Staherb Natural Ingredients Co., Ltd. (Changsha, China). The amount of CGA used here (1.5 g per kg of feed) was in line with the conditions of published studies, and CGA was continually present during the experiment (Liu et al., 2023). An unlimited supply of food and water was made available.

**TABLE 1:** Ingredients and nutrient levels in the basal diet.

Ingredient (g/kg)	Starter (1–21 days)	Grower (22–42 days)
Corn	527.9	577.8
Soybean meal	368.9	300.0
Zea gluten meal	0	24.3
Soybean oil	40.0	40.0
Sodium chloride	3.0	3.0
Choline chloride	3.0	2.6
Vitamin premix^b^	0.3	0.3
Trace element premix^a^	2.0	2.0
Stone powder	12.2	11.7
Dicalcium phosphate	19.1	16.2
DL-Methionine	2.7	1.1
L-Lysine	0.4	0.45
Wheat bran	20.0	20.0
Total	1,000	1,000
Metabolic energy (MJ/kg)	12.4	13.0
Crude protein	211.8	198.4
Lysine	11.4	10.5
Methionine	4.9	4.8
Calcium	10.2	8.5
Available P	4.5	4.2
Total P	6.9	6.3
Threonine	7.7	2.2
Analyzed content		
Calcium	10.2	8.5
Total P	6.8	6.2
Calcium: Total P	1.50	1.37

^a^ Trace element premix is provided as per kg of feed: 8 mg copper (CuSO_4_·5H_2_O); 80 mg iron (FeSO_4_); 100 mg manganese (MnSO_4_·H_2_O); 0.15 mg selenium (Na_2_SeO_3_); 0.35 mg iodine (KI).

^b^ Vitamin premix per kg feed: VA, 9500 IU, VD, 362.5 µg, VE, 30 IU, VK, 32.65 mg, VB1 2 mg, VB6 6 mg, VB12 0.25 mg, biotin 325 μg, folic acid 1.25 mg, pantothenic acid 12 mg, niacin 50 mg.

^c^ Calculated nutrient concentrations.

### 2.4 Sample collection

Two chickens were randomly selected from each replicate and humanely euthanized by inhalation of carbon dioxide. The entire digestive apparatus was swiftly extracted and placed in an icy stainless-steel container and washed with pre-chilled saline solution. The ileum, positioned between Meckel’s diverticulum and the ileocaeco-colic junction, was carefully dissected using sterile forceps and scissors. Approximately 5 cm of the intestine was cut from the middle of the organ and a front portion of ileal approximately 1.5 cm was taken and treated with 4% paraformaldehyde (PFA) for hematoxylin and eosin (H&E) staining and further examination of intestinal structure from 28, 35, 42 days of age, while the remaining part was quickly frozen in liquid nitrogen and stored at −80°C for mRNA expression analysis. At 42 days old, five birds from each treatment were sampled for their ileac contents, which were then immediately placed in a sterile container, frozen with liquid nitrogen, and kept at −80°C for microbial analysis. The entire sampling process was completed in a quarter of an hour.

### 2.5 Intestinal morphology

The tissue samples fixed in 4% PFA were dehydrated with a graded series of ethanol solutions (70%, 96%, and 100%), embedded in paraffin, and 5-μm sections were cut and mounted on a slide. Each section was dewaxed with 100% xylene and then rehydrated. The intestinal tissues were H&E-stained for 8 min, rinsed for 10 s with 1% HCl in ethanol, extensively washed with deionized water, and restrained with eosin for 1 min. Stained slides were washed with deionized water for 6 min, dehydrated with ethanol, cleared in xylene, and allowed to dry overnight. The sections were visualized using a scanner (Pannoramic MIDI, Hungary) for better examination of intestinal morphology and accurately measuring changes in villi. The height of the villi was determined by calculating the distance from the apex to the crypt entrance, and the crypt depth was ascertained by determining the distance from the crypt base to the crypt opening. The average height of villi and depth of crypts was determined by computing the mean values. Case Viewer was used to measure the heights of villi and depths of crypts, and calculate the ratio of villus height to crypt depth (villus height/crypt depth, V/C).

### 2.6 Determination of intestinal mRNA expression by quantitative real-time PCR (qRT-PCR)

Total RNA was isolated from ileal tissues with TRIzol reagent. RNA quality and quantity were established by spectrophotometer (Nanodrop 2000C, Thermo Fisher). The A260/A280 ratio of 1.9-2.0 showed that the RNA was of suitable quality for mRNA determinations. The process of reverse transcribing RNA into cDNA was completed using the universal SYBR qPCR master mix, and a real-time fluorescence quantitative PCR reaction was then performed using the HiScript III RT SuperMix for qPCR kit (Takara Biotechnology Co., Ltd., Tokyo, Japan). The PCR was conducted using a CFX96 thermocycler (Bio-Rad Touch, Bio-Rad) with a 20 μL PCR reaction composed of 2 μL of template cDNA, 10 μL of SYBP master-mix, 0.4 μL of each primer, and 7.2 μL of dd H_2_O. Table 2 provides the primer information of the gene to be tested, while GAPDH was employed as the reference genes. The PCR amplification was followed by the visualization of the melting curve. The PCR amplification was followed by a visualization of the melting curve. The 2^−△△Ct^ relative quantitative method was employed to determine relative gene expression.

**TABLE 2:** Primer sequences of target genes.

Genes^a^	Forward primer (5′-3′)	Reverse primer (5′-3′)	Length	TM^b^°C	Accession No
OCLN	ACG​GCA​GCA​CCT​ACC​TCA​A	GGG​CGA​AGA​AGC​AGA​TGA​G	123	51.7	XM_025144247.2
CLDN1	CAT​ACT​CCT​GGG​TCT​GGT​TGG​T	GACAGCCA TCCGCA TCTTCT	100	51.3	NM_001013611.2
CLDN2	CCT​ACA​TTG​GTT​CAA​GCA​TCG​TGA	GAT​GTC​GGG​AGG​CAG​GTT​GA	131	50.3	NM_001277622.1
ZO-1	CTT​CAG​GTG​TTT​CTC​TTC​CTC​CTC	CTGTGGTTTCA TGGCTGGATC	144	51.5	XM_021098886.1
TNF-α	GAG​CGT​TGA​CTT​GGC​TGT​C	AAGCAACAACCAGCTA TGCAC	176	55.4	NM_214022.1
IL-1β	ACTGGGCA TCAAGGGCTA	GGTAGAAGA TGAAGCGGGTC	154	55.6	NM_214005.1
IL-6	GCTGCGCTTCTACACAGA	TCCCGTTCTCA TCCA TCTTCTC	203	55.4	NM_204628.1
IL-10	AGA​AAT​CCC​TCC​TCG​CCA​AT	AAA​TAG​CGA​ACG​GCC​CTC​A	121	51.2	NM_001004414.2
GAPDH	TGC​TGC​CCA​GAA​CAT​CAT​CC	ACG​GCA​GGT​CAG​GTC​AAC​AA	142	50-60	NM_204305

^a^ Primer sequences of OCLN, CLDN1, CLDN2, ZO-1, TNF-α, IL-1β, IL-6, IL-10, and GAPDH.

^b^ TM, melting temperature.

### 2.7 16s rRNA sequencing of ileal microorganisms

The ileac contents (100 mg) were processed with the QIAamp Fast Stool Mini Kit (Qiagen, Hilden, Germany) to isolate microbial DNA, which was stored at −80°C. The DNA concentration was determined with a Qubit^®^ 3.0 fluorometer, and its integrity was checked by electrophoresis on a 2 % agarose gel. The V3 and V4 hypervariable regions of the 16S rRNA genes were amplified using the primers: 341F—CCTACGGRRBGCASCAGKVRVGAAT, and 806R–GGACTACNVGGGTWTCTAATCC. Adapters were attached to the ends of the amplicons to produce indexed libraries suitable for sequencing on an Illumina Miseq sequencer. The accuracy of the DNA library’s concentration was checked using the Qubit^®^ 3.0 fluorometer. The Illumina MiSeq was loaded with 10 nM DNA libraries which were multiplexed in accordance with the manufacturer’s guidelines (Illumina, San Diego, CA, United States). The raw reads that were acquired were combined into continuous sequences based on the overlaps between them, and any low-quality or inadequate sequences were rejected. The software search (ver 1.9.6) was employed to align the obtained sequences into operational taxonomic units (OTUs) against the Silva 123 database, at a pre-clustered sequence identity of 97%. Venn diagrams were constructed using R (version 3.1.1) in order to highlight the shared and distinct OTUs across the four groups. QIIME (version 1.7.0) was employed to investigate the rarefaction curve, alpha diversity, and beta diversity. The Chao1, Shannon, and Simpson indices revealed the alpha diversity, while principal coordinate analysis (PCoA) was utilized to demonstrate the beta diversity. The non-parametric ANOSIM test was employed to evaluate the differences between groups. The ribosomal database program classifier was applied to assign taxonomic levels down to the genus level, including the kingdom, phylum, class, order, and family, with 80% certainty. The sequence data from our investigation were deposited in the NCBI SRA database (Acc. No. PRJNA916381).

### 2.8 Statistical analysis

The data were analyzed for adherence to a normal distribution using the SPSS statistical package (ver. 20.0 for Windows, SPSS Inc., Chicago, IL, United States). A one-way ANOVA was conducted to compare the data between groups, and Tukey’s multiple comparison procedure was applied when the differences were deemed statistically significant. This statistical analysis only allows for the four treatments to be compared on the same day of the experiment. No discrepancies were observed in the data from different days. The SEM was used to express the results, with *p* < 0.05 being indicative of a significant difference and 0.05 ≤ *p* < 0.1 showing a notable distinction. The figures were created using GraphPad Prism 9 (GraphPad Software Inc., San Diego, CA, UnitedStates).

## 3 Results

### 3.1 Effect of CGA on intestinal morphology of HD chickens

**FIGURE 1:**
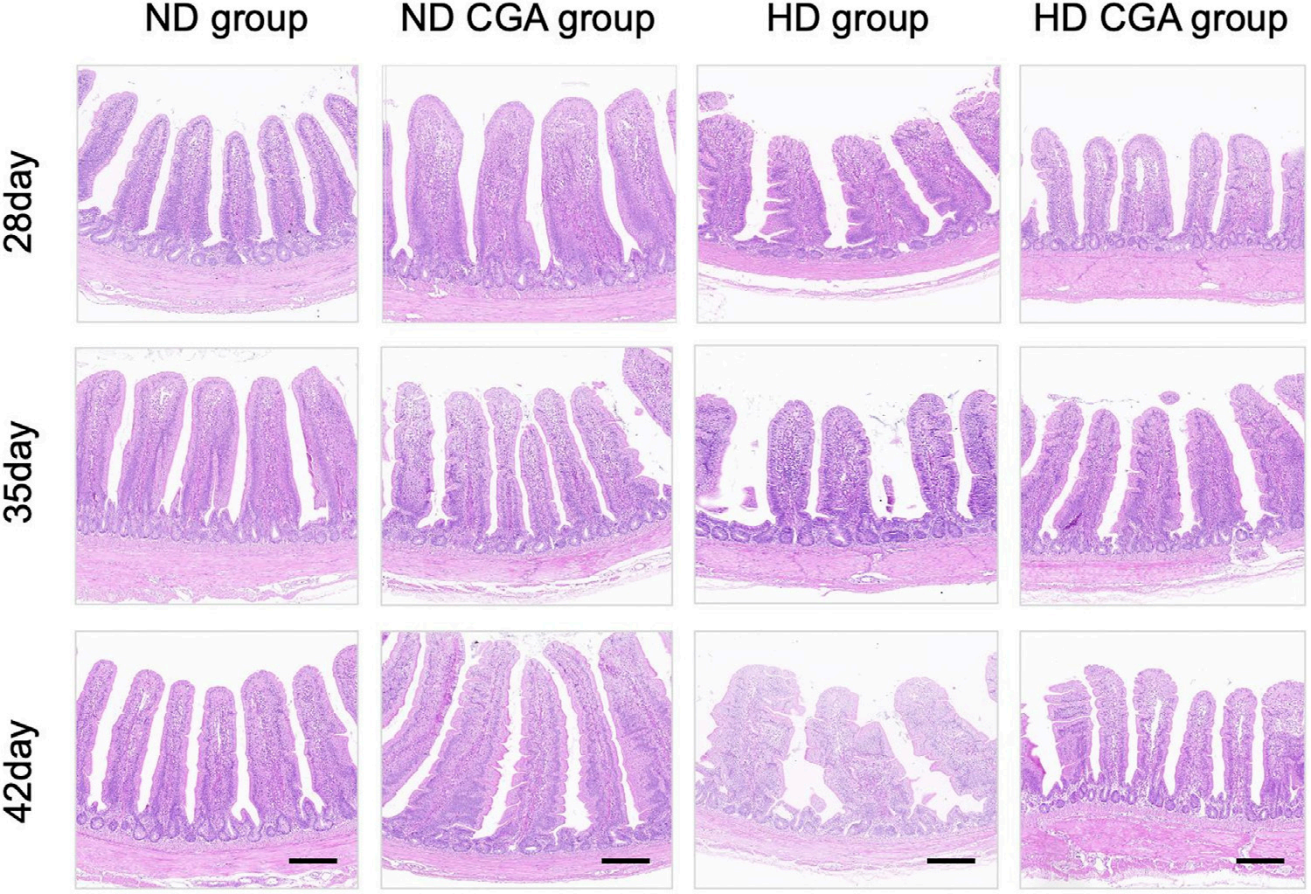
Effects of CGA on ileum morphology of broilers under HD stress. ND group, normal stocking density + basal diet; ND + CGA group, normal stocking density + basal diet +0.15% CGA; HD group, high stocking density + basal diet; HD + CGA group, high stocking density + basal diet +0.15% CGA. Scale bar = 100 µm.

The height of the villi, the crypt depth, and the V to C ratio are shown in Figure 1 and Table 3. On days 28, 35, and 42, the HD broilers had a significantly lower villus height than the NDs (*p* < 0.01), and the ND CGA birds had a higher villus height than the NDs (*p* < 0.01). On days 35 and 42, the HD CGA birds had a higher villus height than the HDs (*p* < 0.01). On days 35 and 42, the V/C was higher in the ND CGA group than in NDs (*p* < 0.01), and on day 42, the V/C was higher in the HD CGA broilers than in the HDs (*p* < 0.01).

**TABLE 3:** Effects of CGA on Ileac morphology of Broilers under HD stress.

Parameter	Days	Dietary treatment^a^				SEM	*p*-value
		ND	ND + CGA	HD	HD + CGA		
VH (µm)	28	747.83^b^	874.31^a^	680.47^c^	725.17^bc^	19.09	<0.01
	35	841.75^b^	942.93^a^	731.68^c^	804.60^b^	19.42	<0.01
	42	893.57^b^	986.23^a^	761.88^c^	881.35^b^	18.09	<0.01
CD (µm)	28	73.90	70.17	84.42	80.88	2.96	0.321
	35	91.18	82.42	96.80	97.98	3.68	0.113
	42	101.38	96.03	111.88	103.17	3.01	0.202
VH/CD	28	7.48	11.50	8.00	8.38	0.59	0.551
	35	9.37^b^	12.22^a^	6.80^b^	8.21^b^	0.59	<0.01
	42	8.98^b^	10.65^a^	6.38^c^	8.40^b^	0.41	<0.01

^a^ ND, group, normal stocking density + basal diet; ND + CGA, group, normal stocking density + basal diet +0.15% CGA; HD, group, high stocking density + basal diet; HD + CGA, group, high stocking density group + basal diet+ 0.15% CGA, group. Values with different letters within the same row are indicative of statistically significant differences (*p* < 0.05, Tukey’s HSD, test after one-way ANOVA). The statistical model used does not allow for a comparison between different days’ data.

### 3.2 Effect of CGA on tight junction gene expression in ileum from HD chickens

**FIGURE 2:**
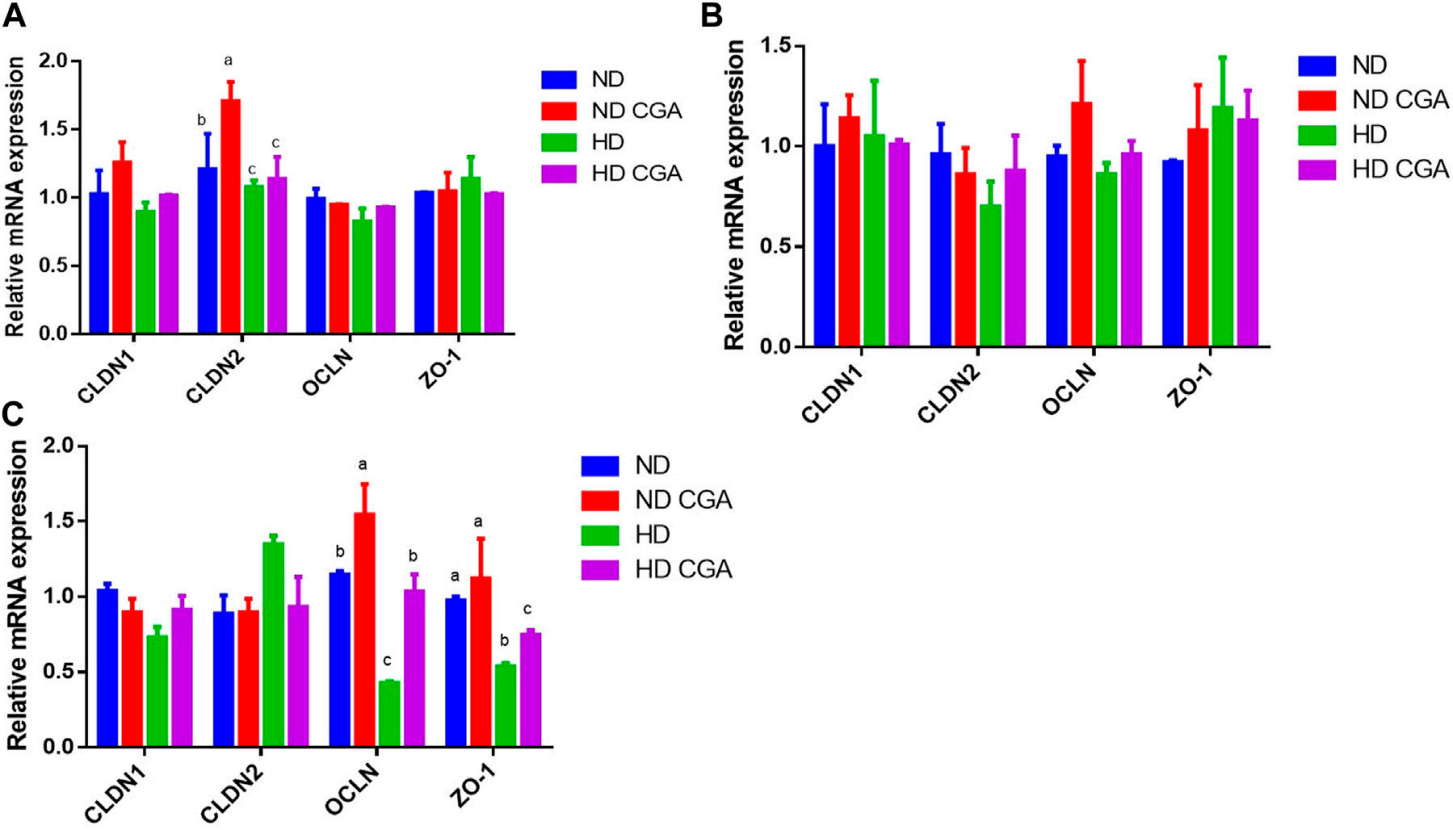
Effects of CGA on ileum mRNA expression of tight junction genes in broilers under HD stress. ND group, normal stocking density + basal diet; ND + CGA group, normal stocking density + basal diet +0.15% CGA; HD group, high stocking density + basal diet; HD + CGA group, high stocking density + basal diet +0.15% CGA. **(A)** Relative mRNA expression at day 28. **(B)** Relative mRNA expression at day 35. **(C)** Relative mRNA expression at day 42. Each vertical bar represents the mean ± SEM (*n* = 10). Values with different letters within the same row are indicative of statistically significant differences (*p* < 0.05, Tukey’s HSD test after one-way ANOVA).

The relative mRNA levels in the ileum of the different groups are shown in Figure 2. The expression of *CLDN-2* was lower (*p* < 0.05) in the HD broilers compared to the ND birds on day 28. The HDs showed a reduced (*p* < 0.05) expression of *OCLN* and *ZO-1* on day 42 when compared to the NDs. On day 28, the ND CGA group had a higher (*p* < 0.05) expression of *CLDN-2* than the ND group. The ND CGA group had a greater *OCLN* expression (*p* < 0.05) than the ND birds on day 42. *OCLN* expression was upregulated (*p* < 0.05) in the HD CGAs compared to the HDs on day 42.

### 3.3 Effect of CGA on inflammatory factors in ileum from HD chickens

**FIGURE 3:**
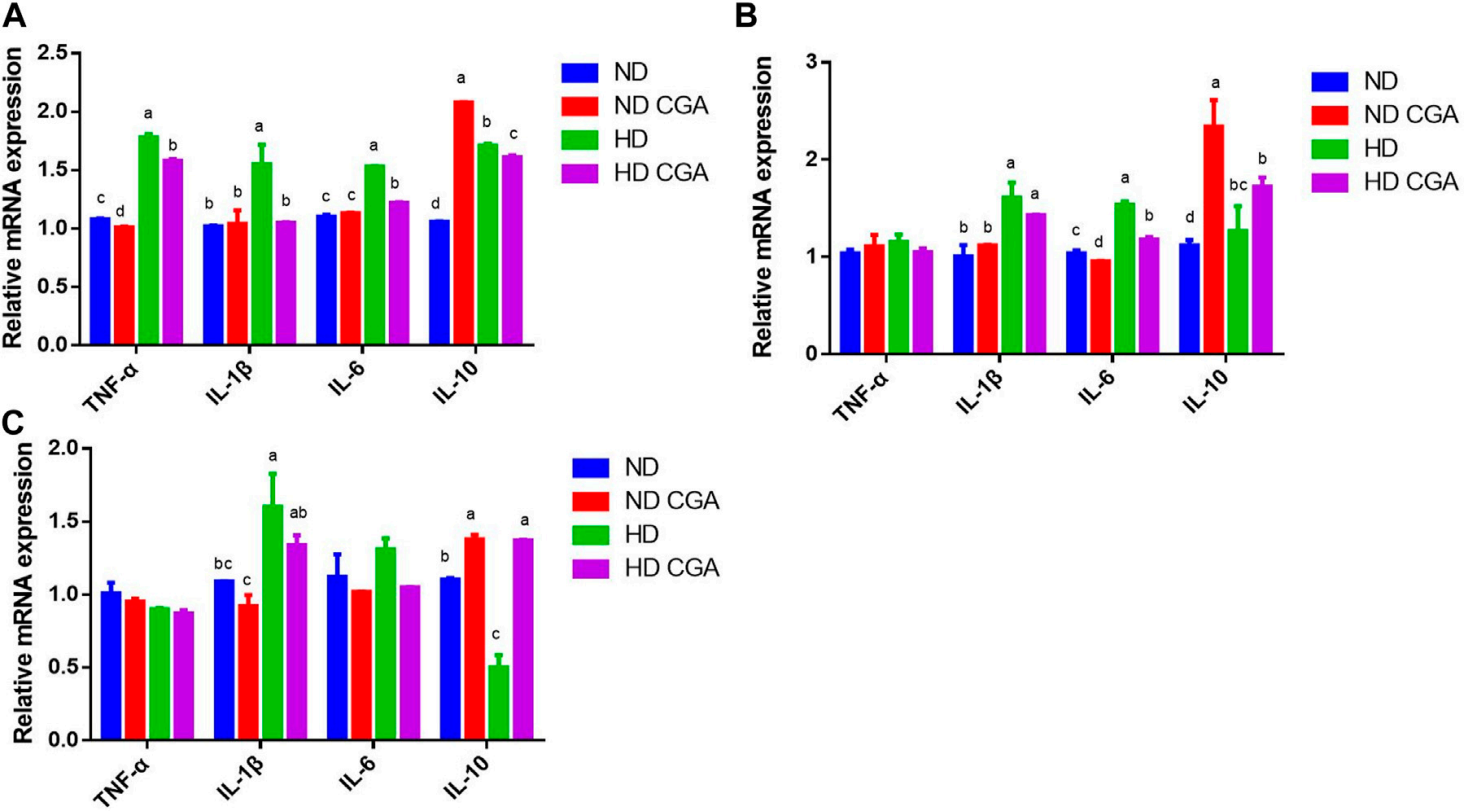
Effects of CGA on ileum mRNA expression of immune factors in broilers under HD stress. ND group, normal stocking density + basal diet; ND + CGA group, normal stocking density + basal diet +0.15% CGA; HD group, high stocking density + basal diet; HD + CGA group, high stocking density + basal diet +0.15% CGA. **(A)** Relative mRNA expression at day 28. **(B)** Relative mRNA expression at day 35. **(C)** Relative mRNA expression at day 42. Each vertical bar represents the mean ± SEM (*n* = 10). Values with different letters within the same row are indicative of statistically significant differences (*p* < 0.05, Tukey’s HSD test after one-way ANOVA).

The mRNA expression of ileal inflammatory cytokines in each group is shown in Figure 3. The expression of *TNF-α*, *IL-1β*, *IL-6*, and *IL-10* was upregulated (*p* < 0.05) in HD broilers compared to NDs at day 28, and decreased (*p* < 0.05) in the HD CGA birds compared to the HDs. The mRNA levels of the inflammatory factor genes, *IL-1β*, *IL-6*, and *IL-10* were significantly upregulated (*p* < 0.05) in HD broilers compared to NDs on day 35, and the expression of *IL-6* was lower (*p* < 0.05) in HD CGA than in HD. *IL-1β* was upregulated (*p* < 0.05) in the HD broilers relative to the NDs on day 42, and the expression of *IL-10* was higher in the HD CGAs (*p* < 0.05) compared to HD chickens without CGA.

### 3.4 Effect of CGA on microbial composition in ileum from HD chickens

**FIGURE 4:**
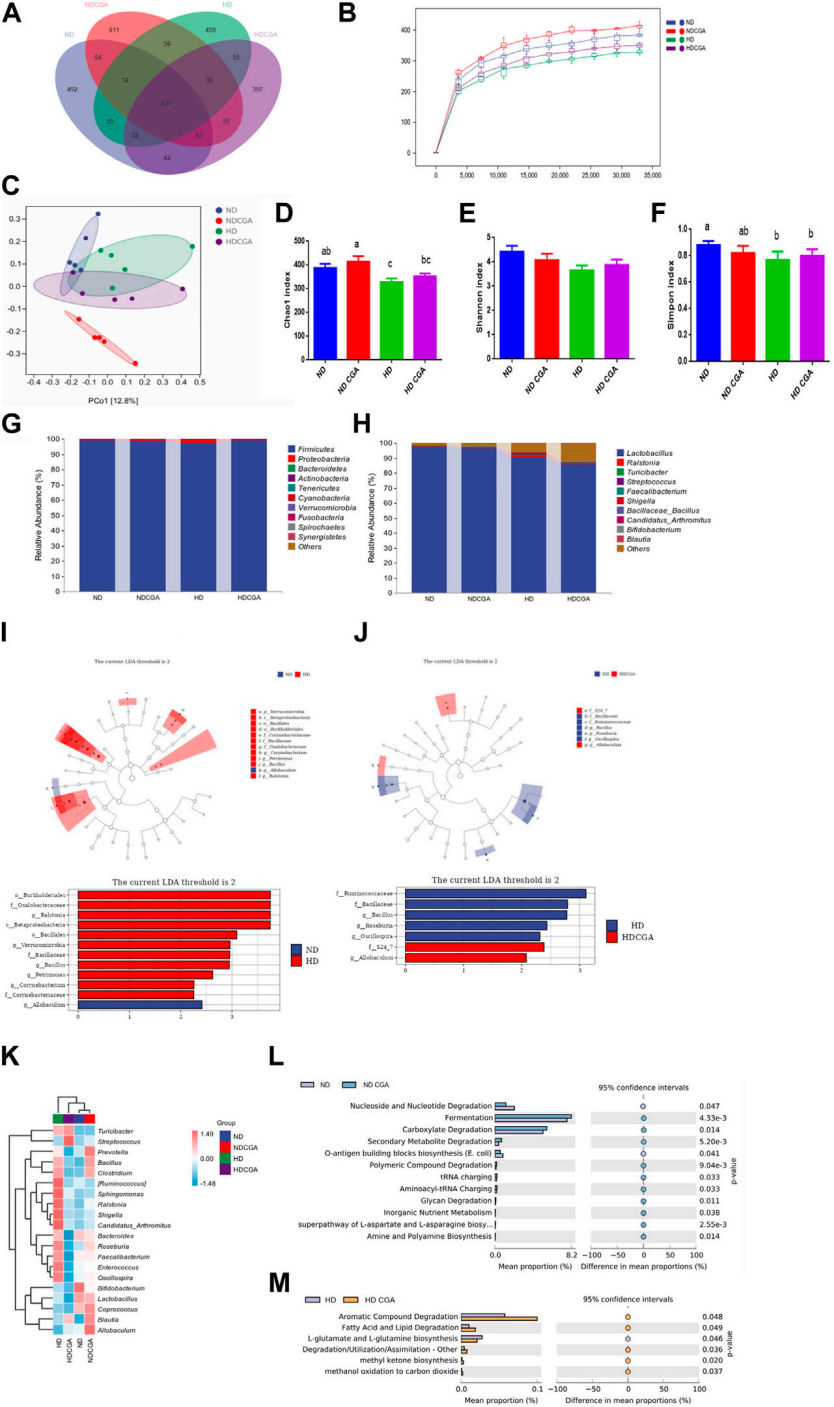
The impact of CGA consumption on the bacterial population in the ileum of chickens from the ND, HD, ND CGA, and HD CGA groups. **(A)** Venn diagram showing unique and shared numbers of genera predicted. **(B)** Rarefaction Curve. **(C)** Two-dimensional OTU abundance based principal coordinate analysis (PCoA) of ileac microbiota. D-F. CGA was found to increase caecal microbial alpha diversity as measured by Simpson and Chao1 indicators. **(G)** Microbial composition at the phylum level. **(H)** Microbial composition at the genus level. **(I-J)** Leaf and bar plots obtained by linear discriminant analysis effect size (LEfSe) analysis showed differences in the abundance of broiler fecal microbes. **(K)** A graphical representation of the range of species present in the top twenty genera in each sample. Pink represents positive correlation and blue indicates negative correlation. **(L-M)** COG functional classification and differences in COG abundance.

Distinctive populations of ileal microorganisms were seen among the four groups (Figure 4). The Venn diagram shows 239 OTUs that were common to all four groups, while the ND, ND CGA, HD, and HD CGA groups were found to contain 1,009, 1,149, 967, and 922 distinct OTUs, respectively. In the ileum of ND, ND CGA, HD, and HD CGA groups, 492, 611, 459, and 397 distinct OTUs were counted, respectively in Figure 4A.

The samples’ species reduction rates are depicted in Figure 4B. The dilution curve can be used to evaluate the accuracy of sequencing data and give an indication of the diversity of species present in the samples. When the sample size from each group exceeds 35,000, the graph tends to level off. The data indicate that the sequencing level is nearly maximized, the greatest diversity is present, and additional data will only yield a few additional species (OTUs). The sequences demonstrate the capacity to accurately portray the microbial population of a natural habitat and can be utilized for data analysis.

The PCoA (Figure 4C) showed that the community composition of the ND group was significantly different from the HD and ND CGA groups, and the difference was quite substantial. In addition, the ND CGA group had a more concentrated distribution than the other two groups. It is evident from Figures 4D–F that the α-diversity index in each group is close to 1, indicating that the data of each group accurately reflect the true ileal microflora composition. The Simpson and Chao bacterial richness and α-diversity indices of HD birds were significantly reduced (*p* < 0.05) compared to ND, as indicated by the decline in single species indices.

Analysis of the taxonomic units indicated that Firmicutes was the dominant phylum among the four groups (Figure 4G; Table 4), making up 99.06%, 98.68%, 97.63%, and 99.00% of the populations, respectively, while Proteobacteria and Bacteroidetes accounted for 0.33%, 0.70%, 1.77%, and 0.55%, and 0.28%, 0.32%, 0.21%, and 0.24%, respectively. The Proteobacteria count for the HD group was significantly higher and the Bacteroidetes count was significantly lower than in NDs (*p* < 0.05). The numbers of Proteobacteria and Bacteroidetes in the ileum of broilers in the HD CGA group was significantly higher than in the HDs (*p* < 0.05). Cyanobacterial abundance was likely affected by CGA, but the difference was statistically non-significant (*p* = 0.057). The predominant genera in the ileum of the four groups as shown in Figure 4H and Table 5 were *Lactobacillus* (97.62%, 96.48%, 90.84%, 86.02%), with minor amounts of *Ralstonia* (0.17%, 0.47%, 1.12%, 0.32%), *Turicibacter* (0.02%, 0.03%, 0.45%, 0.32%), *Streptococcus* (0.12%, 0.11%, 0.28%, 0.16%), *Faecalibacterium* (0.15%, 0.15%, 0.17%, 0.10%), *Shigella* (0.02%, 0.06%, 0.35%, 0.03%), Bacillaceae *Bacillus* (0.02%, 0.20%, 0.18%, 0.05%), *Candidatus arthromitus* (0.02%, 0.07%, 0.30%, 0.06%), *Bifidobacterium* (0.13%, 0.09%, 0.04%, 0.08%), and *Blautia* (0.08%, 0.10%, 0.08%, 0.09%). The ileac *Lactobacillus* and *Bifidobacterium* count in HD birds was significantly lower than those of ND (*p* < 0.05). The HD birds had a significantly larger number of *Turicibacter*, *Streptococcus*, and *Shigella* than the ND (*p* < 0.05). The HD CGA broilers had fewer *Turicibacter*, *Streptococcus*, and *Shigella* compared to the HD group (*p* < 0.05), however, the other dominant bacterial genera were not distinguishable among the four groups.

**TABLE 4:** Composition of ileum microbiota of broilers at phylum level.

Microorganism	Dietary treatment^a^				SEM	*p*-value
	ND	ND + CGA	HD	HD + CGA		
Firmicutes	99.06%	98.68%	97.63%	99.00%	0.012	0.160
Proteobacteria	0.33%^c^	0.70%^b^	1.77%^a^	0.55%^b^	0.011	0.039
Bacteroidetes	0.28%^b^	0.32%^a^	0.21%^b^	0.24%^b^	0.008	0.042
Actinobacteria	0.12%	0.11%	0.17%	0.12%	0.006	0.091
Fusobacteria	0.11%	0.14%	0.13%	0.06%	0.004	0.327
Cyanobacteria	0.01%	0.02%	0.01%	0.01%	0.001	0.057

^a^ ND, group, normal stocking density + basal diet; ND + CGA, group, normal stocking density + basal diet +0.15% CGA; HD, group, high stocking density + basal diet; HD + CGA, group, high stocking density group + basal diet+ 0.15% CGA, group. Values with different letters within the same row are indicative of statistically significant differences (*p* < 0.05, Tukey’s HSD, test after one-way ANOVA).

**TABLE 5:** Composition of ileum microbiota of broilers at genus level.

Microorganism	Dietary treatment^a^				SEM	*p*-value
	ND	ND + CGA	HD	HD + CGA		
*Lactobacillus*	97.62%^a^	96.48%^a^	86.02%^b^	90.84%^b^	0.156	0.046
*Ralstonia*	0.17%	0.47%	1.12%	0.32%	0.008	0.251
*Turicibacter*	0.02%^c^	0.03%^c^	0.45%^a^	0.32%^b^	0.007	0.026
*Streptococcus*	0.12%^b^	0.11%^b^	0.28%^a^	0.16%^b^	0.002	0.047
*Faecalibacterium*	0.15%	0.15%	0.17%	0.10%	0.001	0.256
*Shigella*	0.02%^b^	0.06%^b^	0.35%^a^	0.03%^b^	0.004	0.031
Bacillaceae_*Bacillus*	0.02%	0.20%	0.18%	0.05%	0.001	0.086
*Candidatus_Arthromitus*	0.02%	0.07%	0.30%	0.06%	0.003	0.301
*Bifidobacterium*	0.13%^a^	0.09%^a^	0.04%^b^	0.08%^ab^	0.003	0.035
*Blautia*	0.08%	0.10%	0.08%	0.09%	0.001	0.353

^a^ ND, group, normal stocking density + basal diet; ND + CGA, group, normal stocking density + basal diet +0.15% CGA; HD, group, high stocking density + basal diet; HD + CGA, group, high stocking density group + basal diet+ 0.15% CGA, group. Values with different letters within the same row are indicative of statistically significant differences (*p* < 0.05, Tukey’s HSD, test after one-way ANOVA).

The LEfSe results (Figures 4I, J) indicated that Burkholderiales, Oxalobacteraceae, *Ralstonia*, and Betaproteobacteria were the major bacterial groups present in HD chickens compared to ND. The HD CGA broilers featured *S24-7* and *Allobaculum* as their primary colony members and biomarkers, which was different from the HD group. To illustrate the variation in species and their abundance trends among the groups, the top twenty genera in terms of relative abundance were grouped according to genus and visualized in a heat map (Figure 4K). The ND group was significantly populated with *Bifidobacterium*, *Lactobacillus*, and *Coprococcus*, while *Allobaculum*, *Prevotella*, *Blautia*, and *Coprococcus* were highly represented in ND CGA. The HD birds were populated with *Shigella*, *Ruminococcus*, *C. arthromitus*, and *Sphingomonas*; *Streptococcus*, *Turicibacter*, and *Blautia* were present in increased numbers in HD CGA birds. A comparison between the ND and ND CGA groups revealed that the ND CGA dataset possessed a higher level of annotation richness than the ND (*p* < 0.05), with tRNA charging, fermentation, and degradation of carboxylate, secondary metabolites, and polymeric compounds, being the most common annotations (Figures 4L, M). Comparison between the HD and HD CGA datasets, showed that the HD CGA group had a notably higher number of annotations (*p* < 0.05) than the HD. The most common annotations were aromatic compound degradation, lipid degradation, degradation/utilization/assimilation, methyl ketone biosynthesis, and methanol oxidation to carbon dioxide.

## 4 Discussion

Our previous research has verified that HD stress can significantly hinder the growth and development of broilers, causing physical damage to the jejunum and increasing the level of inflammation, and altering the microbial composition of the jejunum. Nevertheless, supplementing feed with CGA can mitigate the oxidative stress caused by HD stress, thus promoting the growth and development of broilers (Liu et al., 2023). Consequently, the HD stress could lead to damage in other parts of the digestive tract. The structure of the intestines is pivotal in preserving intestinal health (Pourabedin et al., 2014). The small intestine’s digestive and absorptive capabilities can be gauged by looking at the intestinal villus height, crypt depth, and the ratio of villus height to crypt depth (Farahat et al., 2021), which provide a comprehensive evaluation of the organ’s functioning. Therefore, we studied the effects of CGA supplementation on chickens dealing with HD stress by looking at the morphological changes in their intestines over the course of their growth. The results indicated that HD had a more detrimental effect on the villus height of the ileum; nevertheless, CGA was able to lessen the detrimental impact. This suggests that CGA can preserve the healthy development of intestinal villi and avert HD stress. The work of Zhang et al. (2018) demonstrated that inclusion of CGA in the diet of piglets had a beneficial impact on the intestine structure of weaned piglets, leading to a significant increase in small intestinal villus height as well as the V/C ratio of the jejunum and ileum. The study found that CGA supplementation significantly increased the ileum villus height and V/C ratio, and strengthened the intestinal barrier of weaned rats exposed to lipopolysaccharide (Ruan et al., 2014).

The poultry intestine is a complex structure comprised of four distinct parts: physical, chemical, immune, and microbial, that act as a barrier between the animal body and the outside environment (Xiao et al., 2017). The physical barrier acts as a protective shield between the intestinal lumen and the internal environment. When chickens are subjected to stress from crowding, the connections between intestinal jejunal epithelial cells can be weaken, thus allowing a greater number of molecular pathogens and pathogenic bacteria to pass through and stimulate the intestinal immune system (Matricon et al., 2012). Our findings demonstrated that exposure to HD stress caused a considerable decline in the *Occludin* and *ZO-1* mRNA levels in the ileum of broilers, thus disrupting the intestine’s ability to act as a barrier. Many investigations have determined that stress has a detrimental effect on *Occludin* and *ZO-1* in the intestinal mucosa of poultry (Varasteh et al., 2015; Zhang et al., 2017). CGA in the diet of broilers led to a marked increase in *Occludin* and *ZO-1* mRNA levels in the ileum, and successfully averted the disruption of the barrier’s integrity; this shows that CGA has a beneficial effect in reinforcing the mechanical barrier of the intestine. The immune system of the intestinal mucosa is integral to the onset and progression of intestinal inflammation. Cytokines play key roles in an animal’s immune response and defensive mechanisms (Li et al., 2014). An imbalance in the levels of pro-inflammatory and anti-infective cytokines, such as IL-6, IL-1β, and TNF-α in the intestinal mucosal immune system can lead to an impaired immune response and the development of intestinal inflammation (Delgado et al., 2015). Our research has revealed that HD stress can increase intestinal inflammation by altering the expression of inflammatory factors like IL-6, IL-1β, and TNF-α. Another investigation confirmed that HD stress resulted in the induction of the pro-inflammatory cytokines, IL-1β and TNF-α (Bai et al., 2022). Our research indicated that HD stress could alter the intestinal immune balance from an anti-inflammatory to a pro-inflammatory state after 28, 35, and 42 days. Despite this, the incorporation of CGA in the diet was successful in diminishing the presence of inflammatory elements. It appears that CGA has the potential to alleviate inflammation and reduce the pressure of HD.

The intestine not only serves as the site for digestion and nutrient uptake, but is also the body’s most extensive immune organ (Adedokun and Oloejede, 2019). It is essential to sustain a healthy gut for the successful breeding of poultry (Hafez and Attia, 2020). Current research has shown that the composition of the gut microbiome can significantly affect the wellbeing of poultry as well as their productivity (Tan et al., 2010). The intestinal microbial population is an indispensable part of the body, instrumental in maintaining homeostasis not only by facilitating metabolic processes like digestion, nutrient uptake, and energy regulation, but also by preserving the intestinal barrier, and regulating the nervous, endocrine, and immune systems (Ruan et al., 2014).

The present study revealed that CGA supplementation led to a significant increase in microbial diversity in ileac samples from HD-stressed chickens, as demonstrated by the Simpson and Chao1 indices, as well as the PCoA analyses that indicated that the ND CGA group had a dense and uniform bacterial population structure. This type of diversity, referred to as alpha diversity, encompasses the richness, variety, and evenness of species found in a single habitat. Research has demonstrated that CGA has the capability to alter the intestinal microbiota of animals leading to a greater variety of microbial species (Chen et al., 2021). A study found that CGA could counteract the dextran sodium sulfate (DSS)-caused decline in gut microbial diversity in mouse feces, as well as boost the number of *Lactobacillus*, implying that CGA might be able to safeguard the colon from DSS-induced damage by raising the variety of microbes in the gut (Zhang et al., 2019). Research has indicated that Bacteroidetes and Sclerenchyma bacteria located in the digestive system of animals are essential for metabolizing various substances (Ruan et al., 2014). Schreuder et al. (2019) found that the majority of phyla present in the feces of laying hens were Firmicutes and Bacteroidetes. The results of this research demonstrated that, on a phylum level, the gut bacteria were primarily Firmicutes and Proteobacteria. The numbers of Bacteroidetes and Firmicutes fluctuated in response to how effectively energy was acquired from the food consumed, causing an increase in the proportion of Bacteroidetes relative to Sclerenchyma, which could enhance the growth of animals (Turnbaugh et al., 2006). The ND CGA group exhibited a greater prevalence of Bacteroidetes at the phylum level relative to the ND group. Some studies revealed that the inclusion of CGA in the diet of chicken increased Bacteroidetes and decreased Firmicutes in the gut, suggesting that CGA may be able to combat intestinal inflammation (Wang et al., 2019). These conclusions are similar to those found in a previous study (Liu et al., 2023) providing evidence that dietary CGA lessened the impact of HD stress on the gut microbiota of broilers, ultimately leading to better intestinal health.

The presence of *Lactobacillus* in the intestine at the genus level can diminish the adhesiveness of bacterial pathogens like *Escherichia coli* and *Salmonella* to the intestinal wall and improve an animal’s immunity (Wang et al., 2021). Song and others determined that levels of *Lactobacillus* in the digestive tract of chickens were reduced when the birds were stressed (Song et al., 2013). In the current study, HD stress was also seen to reduce the numbers of *Lactobacillus* in the gastrointestinal tract of broilers. The bacterium *Shigella* is the primary cause of bacillary dysentery. A major indication of *Shigella* infection in chickens is the presence of diarrheal stools, as well as ulceration and inflammation of the intestinal lining. Infants and juveniles are particularly vulnerable to *Shigella* infection (Ashkenaz, 2004). Our results demonstrated a large decrease in the prevalence of *Shigella* in HD chickens fed CGA, from 0.35% with no CGA to 0.03% in the HD CGA group, suggesting that CGA can impede the escalation of detrimental bacteria due to HD and boost intestinal health. Bifidobacteria generate a significantly greater amount of lactic and acetic acid than lactobacilli, and bifidobacteria also have a part to play in keeping the gastrointestinal barrier stable, influencing local and systemic immune responses, hindering the penetration of pathogens, and aiding the conversion of indigestible dietary components into beneficial molecules (Cisek and Binek, 2014). Some studies revealed that stress caused a decrease in the numbers of viable lactobacilli and bifidobacteria in the small intestine of broilers (Song et al., 2014). Our research showed that when broilers were subjected to HD, the populations of *Lactobacillus* and *Bifidobacterium* in the ileal digest decreased, while *Streptococcus* and *Shigella* populations increased. The damaging alterations to intestinal microbiota could be a factor in the stress-induced damage to intestinal morphology and permeability associated with raising chickens under HD conditions. *Blautia* may be beneficial in reducing the negative effects of HD stress on the gut microbe populations in chickens because of its capacity for producing short chain fatty acids (SCFAs) (Delgado et al., 2015). In our work, the ND group was found to have a higher prevalence of *Allobaculum* than the HD group, according to LEfse analysis. *Allobaculum* is a genus of Firmicutes that produces high levels of butyrate and efficiently utilizes glucose in the digestive tract. Our data also showed that increased *Allobaculum* levels were associated with higher yields of SCFAs, particularly butyrate (Balakrishnan et al., 2021). In comparison to HD chickens, the HD CGA group was populated with a greater abundance of S24-7 and *Allobaculum*, and these two bacterial species have been linked to the promotion of health (Kumar et al., 2019; Cheng et al., 2021).

The results of PICRUSt’s predictive analysis indicated that the inclusion of CGA in feed can bolster the ability of resident bacteria to ferment or break down carboxylates, degrade secondary metabolites and polymeric compounds, and charge tRNAs. The microorganisms within the digestive tract are essential for carrying out various metabolic processes. Most creatures, including chickens, do not possess enzymes for carbohydrate metabolism, such as glycoside hydrolase, polysaccharide lyase, and carbohydrate esterase (Yeoman et al., 2012). The inability of chickens to process fiber, starch, cellulose, and pectin would require the addition of enzymes to provide adequate nutrition (Jha and Mishra, 2021), but a better way to solve this problem is through the use of gut microbiota. Microorganisms in the intestines aid the animal body in breaking down and assimilating substances that are difficult to digest, creating nutrients that can be utilized (Krajmalnik-Brown et al., 2012). As already noted, some bacteria can ferment carbohydrates to produce short-chain fatty acids, which inhibit pathogens and serve as sources of nourishment and energy for host organisms (den Besten et al., 2013). Many research studies have demonstrated that the host’s capacity to take up ions such as calcium, magnesium and iron is largely enabled by the presence of SCFAs such as acetate, propionate, and butyrate (Yeoman et al., 2012). Consequently, incorporating CGA into broiler feed could enhance the capacity of HD broilers to overcome stress and absorb nutrients to produce energy for growth. Dietary CGA promotes the breakdown of aromatic compounds, fatty acids, and lipids, the production of methyl ketones, and the oxidation of methanol to carbon dioxide as well as other types of degradation, utilization, and assimilation (Upadhyay and Mohan Rao, 2013).The presence of certain bacteria, such as *lactobacilli*, *enterococci*, *bifidobacteria*, *Clostridium* spp. and *Bacteroides* spp. in the gut, can lead to the degradation of bile acids, making it more difficult for fats to be broken down, absorbed, and stored (Begley et al., 2006). In addition, intestinal bacteria also synthesize fatty acids including conjugated linoleic acid (CLA), which is essential for maintaining the health of humans and animals. Research has shown that supplementation with CLA can lead to a heightened level of catalase activity in the liver of chickens, which may be correlated with a decrease in the fat content of the animals (Rahman et al., 2001). To summarize, administering chlorogenic acid to HD-stressed broilers can help optimize fat metabolism and decrease the amount of fat in their bodies.

## 5 Conclusion

**FIGURE 5:**
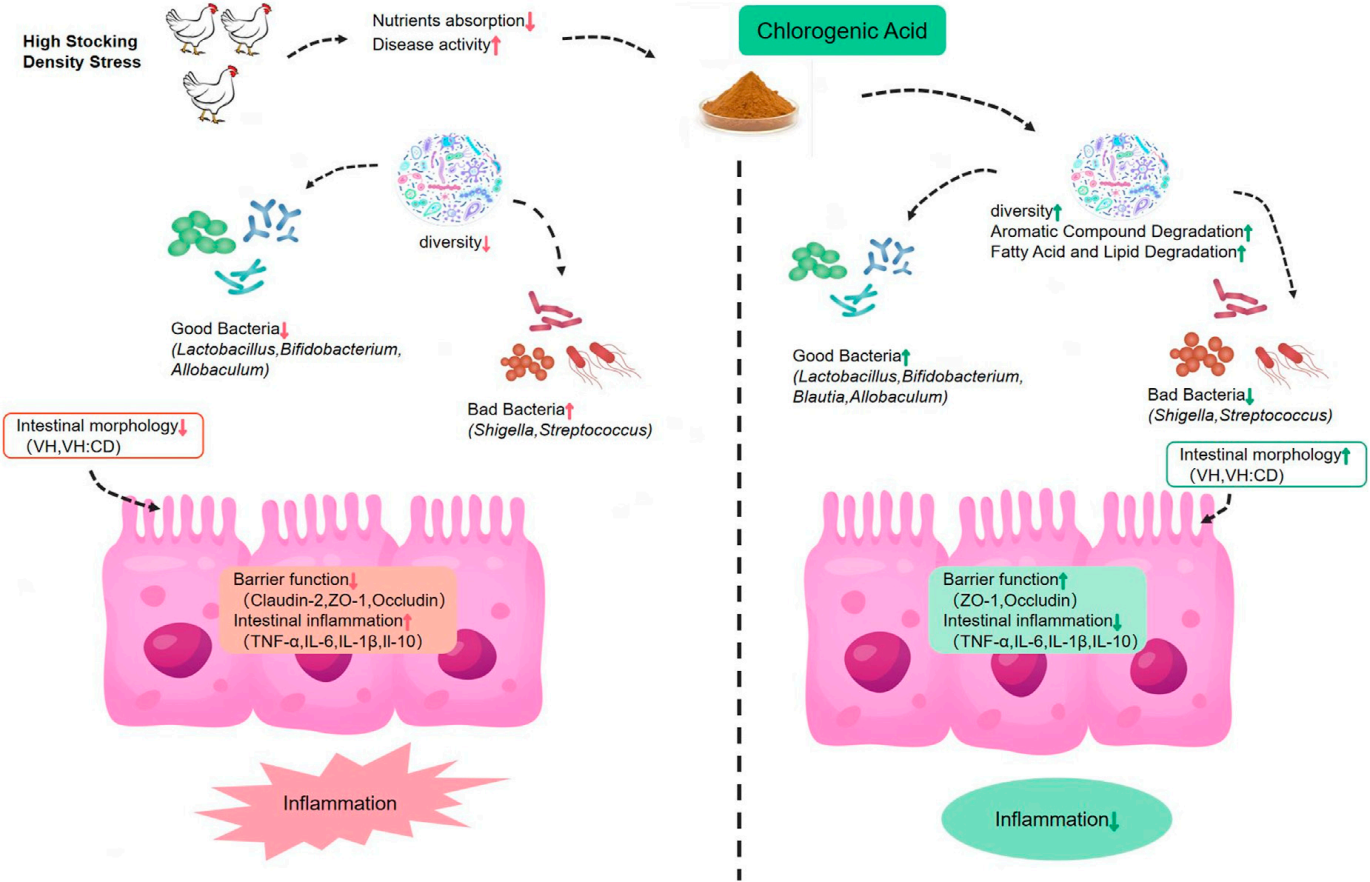
Systematic analysis of the effects of dietary chlorogenic acid on inflammatory index, intestinal barrier function and intestinal microflora in broilers under high stocking density stress.

As depicted in Figure 5, our findings demonstrate that HD stress can result in a reduction of villi size and the V/C ratio in the ileum, decreased expression of tight junction mRNAs, increased expression of pro-inflammatory cytokines, reduced gut microbial diversity, and an increase in the presence of potentially harmful bacteria such as *Turicibacter* and *Shigella*. The supplementation of broiler feed with CGA has been found to enhance the morphology of the ileal intestinal tissue of HD broilers, upregulate the mRNA of tight junction, and reduce the expression of pro-inflammatory cytokines, as well as improve the microbiome composition in the ileum which the phylum-level analysis revealed a decrease in Proteobacteria, and a higher Firmicutes/Bacteroidetes ratio. The presence of *Bacillus* and *Blautia* could be augmented at the genus level, thus allowing for effective regulation of the gut microbiome and improvement in the intestinal health of broilers.

## Data Availability

The datasets presented in this study can be found in online repositories. The names of the repository/repositories and accession number(s) can be found below: https://www.ncbi.nlm.nih.gov/, No. PRJNA916381.

## Abbreviations

CD, ileum crypt depth; CGA, chlorogenic acid; CLA, conjugated linoleic acid; CLDN1, Claudin-1; CLDN2, Claudin-2; GAPDH, glyceraldehyde-3-phosphate dehydrogenase; GSH-Px, glutathione peroxidase; HD, high density stocking; HD CGA, HD+CGA group; H&E, hematoxylin and eosin staining; IL-1β, interleukin-1β; IL-6, interleukin-6; IL-10, interleukin-10; LEfSe, LDA effect size; ND, normal density stocking; ND CGA, ND+CGA group; OCLN, occludin; OUT, operational taxonomic unit; PCoA, principal coordinates analysis; PFA, paraformaldehyde; SCFAs, short-chain fatty acids; TM, melting temperature; TNF-α, tumornecrosis factor-alpha; TRIZOL, total RNA extraction reagent; VH, ileum villus height; VH/CD, ratio of ileum villus height to ileum crypt depth; ZO-1, zonula occludens-1.

## References

[B1] Abd El-Hack M. E. Abdelnour S. A. Taha A. E. Khafaga A. F. Arif M. Ayasan T. (2020). Herbs as thermoregulatory agents in poultry: An overview. Sci. Total Environ. 703, 134399. 10.1016/j.scitotenv.2019.134399 31757531

[B2] Adedokun S. A. Olojede O. C. (2019). Optimizing gastrointestinal integrity in poultry: The role of nutrients and feed additives. Front. Vet. Sci. 5, 348. 10.3389/fvets.2018.00348 30766877 PMC6366008

[B3] Ashkenazi S. (2004). Shigella infections in children: New insights. Semin. Pediatr. Infect. Dis. 15, 246–252. 10.1053/j.spid.2004.07.005 15494948

[B4] Bai D. Liu K. He X. Tan H. Liu Y. Li Y. (2022). Effect of dietary chlorogenic acid on growth performance, antioxidant function, and immune response of broiler breeders under immune stress and stocking density stress. Vet. Sci. 9 (10), 582. 10.3390/vetsci9100582 36288195 PMC9611266

[B5] Balakrishnan B. Luckey D. Bodhke R. Chen J. Marietta E. Jeraldo P. (2021). Prevotella histicola protects from arthritis by expansion of Allobaculum and augmenting butyrate production in humanized mice. Front. Immunol. 12, 609644. 10.3389/fimmu.2021.609644 34017324 PMC8130672

[B6] Begley M. Hill C. Gahan C. G. (2006). Bile salt hydrolase activity in probiotics. Appl. Environ. Microbiol. 72, 1729–1738. 10.1128/AEM.72.3.1729-1738.2006 16517616 PMC1393245

[B7] Chen F. Zhang H. Zhao N. Yang X. Du E. Huang S. (2021). Effect of chlorogenic acid on intestinal inflammation, antioxidant status, and microbial community of young hens challenged with acute heat stress. Anim. Sci. J. 92, e13619. 10.1111/asj.13619 34409681

[B8] Cheng L. Zhang W. Jin Q. Zhu Y. Chen R. Tian Q. (2021). The effects of dietary supplementation with lotus leaf extract on the immune response and intestinal microbiota composition of broiler chickens. Poult. Sci. 100 (3), 100925. 10.1016/j.psj.2020.12.023 33518323 PMC7936220

[B9] Cisek A. A. Binek M. (2014). Chicken intestinal microbiota function with a special emphasis on the role of probiotic bacteria. Pol. J. Vet. Sci. 17, 385–394. 10.2478/pjvs-2014-0057 24988871

[B10] Delgado S. Leite A. M. Ruas-Madiedo P. Mayo B. (2015). Probiotic and technological properties of Lactobacillus spp. strains from the human stomach in the search for potential candidates against gastric microbial dysbiosis. Front. Microbiol. 5, 766. 10.3389/fmicb.2014.00766 25642213 PMC4294198

[B11] den Besten G. van Eunen K. Groen A. K. Venema K. Reijngoud D. J. Bakker B. M. (2013). The role of short-chain fatty acids in the interplay between diet, gut microbiota, and host energy metabolism. J. Lipid Res. 54 (9), 2325–2340. 10.1194/jlr.R036012 23821742 PMC3735932

[B12] Dozier W. A. 3rd Thaxton J. P. Branton S. L. Morgan G. W. Miles D. M. Roush W. B. (2005). Stocking density effects on growth performance and processing yields of heavy broilers. Poult. Sci. 84, 1332–1338. 10.1093/ps/84.8.1332 16156220

[B13] Farahat M. Ibrahim D. Kishawy A. T. Y. Abdallah H. M. Hernandez-Santana A. Attia G. (2021). Effect of cereal type and plant extract addition on the growth performance, intestinal morphology, caecal microflora, and gut barriers gene expression of broiler chickens. Animal 15, 100056. 10.1016/j.animal.2020.100056 33573933

[B14] Gamaleldin Elsadig Karar M. Matei M. F. Jaiswal R. Illenberger S. Kuhnert N. (2016). Neuraminidase inhibition of Dietary chlorogenic acids and derivatives - potential antivirals from dietary sources. Food Funct. 7, 2052–2059. 10.1039/c5fo01412c 27010419

[B15] Hafez H. M. Attia Y. A. (2020). Challenges to the poultry industry: Current perspectives and strategic future after the COVID-19 outbreak. Front. Vet. Sci. 7, 516. 10.3389/fvets.2020.00516 33005639 PMC7479178

[B16] He S. Liu F. Xu L. Yin P. Li D. Mei C. (2016). Protective effects of ferulic acid against heat stress-induced intestinal epithelial barrier dysfunction *in vitro* and *in vivo* . PLoS One 11, e0145236. 10.1371/journal.pone.0145236 26894689 PMC4760716

[B17] He S. Yu Q. He Y. Hu R. Xia S. He J. (2019). Dietary resveratrol supplementation inhibits heat stress-induced high-activated innate immunity and inflammatory response in spleen of yellow-feather broilers. Poult. Sci. 98, 6378–6387. 10.3382/ps/pez471 31406997 PMC8913767

[B18] Jha R. Mishra P. (2021). Dietary fiber in poultry nutrition and their effects on nutrient utilization, performance, gut health, and on the environment: A review. J. Anim. Sci. Biotechnol. 12 (1), 51. 10.1186/s40104-021-00576-0 33866972 PMC8054369

[B19] Jiang J. Xiong Y. L. (2016). Natural antioxidants as food and feed additives to promote health benefits and quality of meat products: A review. Meat Sci. 120, 107–117. 10.1016/j.meatsci.2016.04.005 27091079

[B20] Krajmalnik-Brown R. Ilhan Z. E. Kang D. W. DiBaise J. K. (2012). Effects of gut microbes on nutrient absorption and energy regulation. Nutr. Clin. Pract. 27 (2), 201–214. 10.1177/0884533611436116 22367888 PMC3601187

[B21] Kumar S. Shang Y. Kim W. K. (2019). Insight into dynamics of gut microbial community of broilers fed with fructooligosaccharides supplemented low calcium and phosphorus diets. Front. Vet. Sci. 6, 95. 10.3389/fvets.2019.00095 30984773 PMC6449842

[B22] Leiss K. A. Maltese F. Choi Y. H. Verpoorte R. Klinkhamer P. G. (2009). Identification of chlorogenic acid as a resistance factor for thrips in chrysanthemum. Plant Physiol. 150, 1567–1575. 10.1104/pp.109.138131 19448039 PMC2705022

[B23] Li W. Wei F. Xu B. Sun Q. Deng W. Ma H. (2019). Effect of stocking density and alpha-lipoic acid on the growth performance, physiological and oxidative stress and immune response of broilers. Asian-Australas J. Anim. Sci. 32, 1914–1922. 10.5713/ajas.18.0939 31010966 PMC6819680

[B24] Li Y. Ma Q. G. Zhao L. H. Wei H. Duan G. X. Zhang J. Y. (2014). Effects of lipoic acid on immune function, the antioxidant defense system, and inflammation-related genes expression of broiler chickens fed aflatoxin contaminated diets. Int. J. Mol. Sci. 15 (4), 5649–5662. 10.3390/ijms15045649 24699046 PMC4013587

[B25] Liang N. Kitts D. D. (2015). Role of chlorogenic acids in controlling oxidative and inflammatory stress conditions. Nutrients 8, 16. 10.3390/nu8010016 26712785 PMC4728630

[B26] Liu Y. Zhang Y. Bai D. Li Y. He X. Ito K. (2023). Dietary supplementation with chlorogenic acid enhances antioxidant capacity, which promotes growth, jejunum barrier function, and cecum microbiota in broilers under high stocking density stress. Animals 13, 303. 10.3390/ani13020303 36670842 PMC9854556

[B27] Matricon J. Meleine M. Gelot A. Piche T. Dapoigny M. Muller E. (2012). Review article: Associations between immune activation, intestinal permeability and the irritable bowel syndrome. Aliment. Pharmacol. Ther. 36, 1009–1031. 10.1111/apt.12080 23066886

[B28] Nasr M. A. F. Alkhedaide A. Q. Ramadan A. A. I. Hafez A. S. E. Hussein M. A. (2021). Potential impact of stocking density on growth, carcass traits, indicators of biochemical and oxidative stress and meat quality of different broiler breeds. Poult. Sci. 100, 101442. 10.1016/j.psj.2021.101442 34607150 PMC8493580

[B29] Pourabedin M. Xu Z. Baurhoo B. Chevaux E. Zhao X. (2014). Effects of mannan oligosaccharide and virginiamycin on the cecal microbial community and intestinal morphology of chickens raised under suboptimal conditions. Can. J. Microbiol. 60 (5), 255–266. 10.1139/cjm-2013-0899 24766220

[B30] Quinteiro-Filho W. M. Calefi A. S. Cruz D. S. G. Aloia T. P. A. Zager A. Astolfi-Ferreira C. S. (2017). Heat stress decreases expression of the cytokines, avian β-defensins 4 and 6 and Toll-like receptor 2 in broiler chickens infected with Salmonella Enteritidis. Vet. Immunol. Immunopathol. 186, 19–28. 10.1016/j.vetimm.2017.02.006 28413046

[B31] Rahman S. M. Wang Y. Yotsumoto H. Cha J. Han S. Inoue S. (2001). Effects of conjugated linoleic acid on serum leptin concentration, body-fat accumulation, and beta-oxidation of fatty acid in OLETF rats. Nutrition 17, 385–390. 10.1016/s0899-9007(00)00584-0 11377131

[B32] Ruan Z. Liu S. Zhou Y. Mi S. Liu G. Wu X. (2014). Chlorogenic acid decreases intestinal permeability and increases expression of intestinal tight junction proteins in weaned rats challenged with LPS. PLoS One 9 (6), e97815. 10.1371/journal.pone.0097815 24887396 PMC4041575

[B33] Schreuder J. Velkers F. C. Bouwstra R. J. Beerens N. Stegeman J. A. de Boer W. F. (2019). Limited changes in the fecal microbiome composition of laying hens after oral inoculation with wild duck feces. Poult. Sci. 98, 6542–6551. 10.3382/ps/pez526 31541252 PMC8913958

[B34] Simitzis P. E. Kalogeraki E. Goliomytis M. Charismiadou M. A. Triantaphyllopoulos K. Ayoutanti A. (2012). Impact of stocking density on broiler growth performance, meat characteristics, behavioural components and indicators of physiological and oxidative stress. Br. Poult. Sci. 53, 721–730. 10.1080/00071668.2012.745930 23398415

[B35] Song J. Jiao L. F. Xiao K. Luan Z. Hu C. Shi B. (2013). Cello-oligosaccharide ameliorates heat stress-induced impairment of intestinal microflora, morphology and barrier integrity in broilers. Anim Feed Sci Technol 185 (3-4), 175–181. 10.1016/j.anifeedsci.2013.08.001

[B36] Song J. Xiao K. Ke Y. L. Jiao L. F. Hu C. H. Diao Q. Y. (2014). Effect of a probiotic mixture on intestinal microflora, morphology, and barrier integrity of broilers subjected to heat stress. Poult. Sci. 93(3), 581–588. 10.3382/ps.2013-03455 24604851

[B37] Stanley D. Hughes R. J. Moore R. J. (2014). Microbiota of the chicken gastrointestinal tract: Influence on health, productivity and disease. Appl. Microbiol. Biotechnol. 98, 4301–4310. 10.1007/s00253-014-5646-2 24643736

[B38] Sugiharto S. (2022). Dietary strategies to alleviate high-stocking-density-induced stress in broiler chickens - a comprehensive review. Arch. Anim. Breed. 65 (1), 21–36. 10.5194/aab-65-21-2022 35106363 PMC8795885

[B39] Sung Y. Y. Kim D. S. Kim H. K. (2015). Akebia quinata extract exerts anti-obesity and hypolipidemic effects in high-fat diet-fed mice and 3T3-L1 adipocytes. *J. Ethnopharmacol*. 168, 17–24. 10.1016/j.jep.2015.03.051 25835369

[B40] Tajik N. Tajik M. Mack I. Enck P. (2017). The potential effects of chlorogenic acid, the main phenolic components in coffee, on health: A comprehensive review of the literature. Eur. J. Nutr. 56, 2215–2244. 10.1007/s00394-017-1379-1 28391515

[B41] Tan G. Y. Yang L. Fu Y. Q. Feng J. H. Zhang M. H. (2010). Effects of different acute high ambient temperatures on function of hepatic mitochondrial respiration, antioxidative enzymes, and oxidative injury in broiler chickens. Poult. Sci. 89, 115–122. 10.3382/ps.2009-00318 20008809

[B42] Thaxton J. P. Dozier W. A. 3rd Branton S. L. Morgan G. W. Miles D. W. Roush W. B. (2006). Stocking density and physiological adaptive responses of broilers. Poult. Sci. 85, 819–824. 10.1093/ps/85.5.819 16673757

[B43] Turnbaugh P. J. Ley R. E. Mahowald M. A. Magrini V. Mardis E. R. Gordon J. I. (2006). An obesity-associated gut microbiome with increased capacity for energy harvest. Nature 444, 1027–1031. 10.1038/nature05414 17183312

[B44] Upadhyay R. Mohan Rao L. J. (2013). An outlook on chlorogenic acids-occurrence, chemistry, technology, and biological activities. Crit. Rev. Food Sci. Nutr. 53 (9), 968–984. 10.1080/10408398.2011.576319 23768188

[B45] Vanhonacker F. Verbeke W. Van. Poucke E. Buijs S. Yuyttens A. F. (2009). Societal concern related to stocking density, pen size and group size in farm animal production. Livest. Sci. 123, 16–22. 10.1016/j.livsci.2008.09.023

[B46] Varasteh S. Braber S. Akbari P. Garssen J. Fink-Gremmels J. (2015). Differences in susceptibility to heat stress along the chicken intestine and the protective effects of galacto-Oligosaccharides. PLoS One 10, e0138975. 10.1371/journal.pone.0138975 26402906 PMC4581695

[B47] Vizzier Thaxton Y. Christensen K. D. Mench J. A. Rumley E. R. Daugherty C. Feinberg B. (2016). Symposium: Animal welfare challenges for today and tomorrow. Poult. Sci. 95, 2198–2207. 10.3382/ps/pew099 26994205

[B48] Vukelić I. Detel D. Pučar L. B. Potočnjak I. Buljević S. Domitrović R. (2018). Chlorogenic acid ameliorates experimental colitis in mice by suppressing signaling pathways involved in inflammatory response and apoptosis. Food Chem. Toxicol. 121, 140–150. 10.1016/j.fct.2018.08.061 30165128

[B50] Wang J. Ishfaq M. Li J. (2021). Lactobacillus salivarius ameliorated mycoplasma gallisepticum-induced inflammatory injury and secondary *Escherichia coli* infection in chickens: Involvement of intestinal microbiota. Vet. Immunol. Immunopathol. 233, 110192. 10.1016/j.vetimm.2021.110192 33476924

[B51] Wang W. W. Jia H. J. Zhang H. J. Wang J. Lv H. Y. Wu S. G. (2019). Supplemental plant extracts from flos lonicerae in combination with baikal skullcap attenuate intestinal disruption and modulate gut microbiota in laying hens challenged by salmonella pullorum. Front. Microbiol. 10, 1681. 10.3389/fmicb.2019.01681 31396190 PMC6668501

[B52] Xiao Y. Xiang Y. Zhou W. Chen J. Li K. Yang H. (2017). Microbial community mapping in intestinal tract of broiler chicken. Poult. Sci. 96, 1387–1393. 10.3382/ps/pew372 28339527

[B54] Yeoman C. J. Chia N. Jeraldo P. Sipos M. Goldenfeld N. D. White B. A. (2012). The microbiome of the chicken gastrointestinal tract. Anim. Health Res. Rev. 13, 89–99. 10.1017/S1466252312000138 22853945

[B55] Zhang C. Zhao X. H. Yang L. Chen X. Y. Jiang R. S. Jin S. H. (2017). Resveratrol alleviates heat stress-induced impairment of intestinal morphology, microflora, and barrier integrity in broilers. Poult. Sci. 96, 4325–4332. 10.3382/ps/pex266 29053872

[B56] Zhang P. Jiao H. Wang C. Lin Y. You S. (2019). Chlorogenic acid ameliorates colitis and alters colonic microbiota in a mouse model of dextran sulfate sodium-induced colitis. Front. Physiol. 10, 325. 10.3389/fphys.2019.00325 30971953 PMC6446884

[B57] Zhang X. Zhao Q. Ci X. Chen S. Xie Z. Li H. (2020). Evaluation of the efficacy of chlorogenic acid in reducing small intestine injury, oxidative stress, and inflammation in chickens challenged with *Clostridium perfringens* type A. Poult. Sci. 99, 6606–6618. 10.1016/j.psj.2020.09.082 33248576 PMC7810911

[B58] Zhang Y. Wang Y. Chen D. Yu B. Zheng P. Mao X. (2018). Dietary chlorogenic acid supplementation affects gut morphology, antioxidant capacity and intestinal selected bacterial populations in weaned piglets. Food Funct. 9, 4968–4978. 10.1039/c8fo01126e 30183786

